# Soluble HLA-G Expression Inversely Correlates With Fetal Microchimerism Levels in Peripheral Blood From Women With Scleroderma

**DOI:** 10.3389/fimmu.2018.01685

**Published:** 2018-08-14

**Authors:** Julie Di Cristofaro, Karlin R. Karlmark, Sami B. Kanaan, Doua F. Azzouz, Marina El Haddad, Lucas Hubert, Dominique Farge-Bancel, Brigitte Granel, Jean Robert Harlé, Eric Hachulla, Etienne Pardoux, Jean Roudier, Christophe Picard, Nathalie C. Lambert

**Affiliations:** ^1^Aix Marseille Univ, CNRS, EFS, ADES, “Biologie des Groupes Sanguins”, Marseille, France; ^2^Aix Marseille Univ, INSERM, Autoimmune Arthritis (AA), Marseille, France; ^3^Immunogenetics Laboratory, EFS-Alpes Méditerranée, Marseille, France; ^4^Antibody Therapeutics and Immunotargeting, CRCM, INSERM U1068, Institut Paoli Calmettes, Aix-Marseille Université, Marseille, France; ^5^UM 105, CNRS UMR7258, Marseille, France; ^6^Unité de Médecine Interne Maladies Auto-immunes et Pathologie Vasculaire (UF 04) Hôpital Saint Louis, AP-HP, Centre de Référence des Maladies auto-immunes systémiques Rares d’Île-de-France, FAI2R, EA 3518, Institut Universitaire d’Hématologie, Paris, France; ^7^UMR-S 1076 Endothélium, Pathologies Vasculaires et Cibles Thérapeutiques – Faculté de Pharmacie, Marseille, France; ^8^AP-HM, Pôle de Médecine Interne, Centre de Compétence PACA Ouest pour la prise en charge des maladies autoimmunes systémiques, Marseille, France; ^9^Service de Médecine Interne, Centre National de Référence de la Sclérodermie Systémique, Hôpital Claude Huriez, Lille, France; ^10^Aix Marseille Univ, CNRS, Centrale Marseille, I2M, Marseille, France; ^11^Rhumatologie, IML, AP-HM, Hôpital Sainte Marguerite, Marseille, France

**Keywords:** human leukocyte antigen-G, microchimerism, scleroderma, systemic sclerosis, pregnancy, fetal

## Abstract

Women with scleroderma (SSc) maintain significantly higher quantities of persisting fetal microchimerism (FMc) from complete or incomplete pregnancies in their peripheral blood compared to healthy women. The non-classical class-I human leukocyte antigen (HLA) molecule HLA-G plays a pivotal role for the implantation and maintenance of pregnancy and has often been investigated in offspring from women with pregnancy complications. However data show that maternal *HLA-G* polymorphisms as well as maternal soluble HLA-G (sHLA-G) expression could influence pregnancy outcome. Here, we aimed to investigate the underlying role of maternal sHLA-G expression and *HLA-G* polymorphisms on the persistence of FMc. We measured sHLA-G levels by enzyme linked immunosorbent assay in plasma samples from 88 healthy women and 74 women with SSc. Male Mc was quantified by DYS14 real-time PCR in blood samples from 58 women who had previously given birth to at least one male child. Furthermore, eight *HLA-G* 5′URR/3′UTR polymorphisms, previously described as influencing HLA-G expression, were performed on DNA samples from 96 healthy women and 106 women with SSc. Peripheral sHLA-G was at lower concentration in plasma from SSc (76.2 ± 48.3 IU/mL) compared to healthy women (117.5 ± 60.1 IU/mL, *p* < 0.0001), independently of clinical subtypes, autoantibody profiles, disease duration, or treatments. Moreover, sHLA-G levels were inversely correlated to FMc quantities (Spearman correlation, *p* < 0.01). Finally, women with SSc had lower sHLA-G independently of the eight *HLA-G* 5′URR/3′UTR polymorphisms, although they were statistically more often homozygous than heterozygous for *HLA-G* polymorphism genotypes −716 (G/T), −201 (G/A), 14 bp (ins/del), and +3,142 (G/A) than healthy women. In conclusion, women with SSc display less sHLA-G expression independently of the eight *HLA-G* polymorphisms tested. This decreased production correlates with higher quantities of persisting FMc commonly observed in blood from SSc women. These results shed some lights on the contribution of the maternal HLA-G protein to long-term persistent fetal Mc and initiate new perspectives in this field.

## Introduction

Scleroderma is a rare, invalidating, and complex autoimmune disease, manifested by vascular abnormalities, extensive collagen deposition in the skin, and fibrotic changes in internal organs. SSc is divided into two forms: limited cutaneous disease (lcSSc) characterized by skin involvement below the elbows and knees and diffuse cutaneous disease (dcSSc) for which skin involvement is more extended, with respectively anti-centromere or anti-topoisomerase antibodies (ACA or ATA) as hallmark of each subset (1).

SSc has a strong predilection for women with a peak incidence in women after childbearing years. Moreover, SSc shares some clinical similarities to chronic graft-vs-host disease (cGVHD) (2, 3). Both diseases are characterized by skin, lung, and esophageal involvement and intense fibrosis (2). In cGvHD, donor cells attack the recipient after allogeneic bone marrow stem cell transplantation. This led to the hypothesis that fetal microchimerism (FMc) arising from pregnancy and persisting at long term in the mother may act against the “host” in an “auto/allo” immune reaction and be involved in the development of SSc [for reviews see Ref. (4, 5)].

Although only few studies have demonstrated a role for microchimeric fetal cells in SSc (6–8), many have shown higher levels of fetal Mc in peripheral blood mononuclear cells from women with SSc compared to healthy women (6, 9–12). A few propositions have been raised to explain the possible genetic susceptibility for higher passage of fetal cells during pregnancy and/or maintenance of post-delivery in women with SSc. The host’s and/or donor’s Human Leukocyte Antigen (HLA) genotype may influence the likelihood of having microchimeric cells in host’s blood as previously suggested in SSc and juvenile dermatomyosis (13, 14).

A non-classical class-I HLA molecule, HLA-G, described to play a pivotal role for the implantation and maintenance of pregnancy [for review see Ref. (15)], could influence fetal Mc. As the expression of cell-associated and secreted HLA-G antigens was first and mostly described in placenta on villous cytotrophoblasts at the feto–maternal interface (16, 17), HLA-G has often been investigated in offspring from women with pregnancy complications. Fetal HLA-G is the specific ligand of some receptors (i.e., ILT2, ILT4, and KIR2DL4) present on maternal decidual NK, dendritic cells, and macrophages, which constitute a large part of the immune cells in the uterine compartment, and exerts immunomodulatory functions to secure acceptance of the semiallogenic fetus [for review see Ref. (18)]. Nevertheless, maternal monocytes and dendritic cells express membrane-bound and soluble HLA-G (sHLA-G), respectively (19). The role of maternal HLA-G on the immune tolerance process in pregnancy is poorly understood, although data show that maternal *HLA-G* polymorphisms as well as maternal sHLA-G expression could influence pregnancy outcome (20, 21).

Interestingly, women with pregnancy complications have lower sHLA-G concentration in their plasma (22, 23) and higher fetal Mc passage in their circulation than women with healthy pregnancies (24, 25). Moreover, women with pregnancy complications are at higher risk to develop later autoimmune diseases, such as scleroderma or rheumatoid arthritis (26–28). This has drawn our attention on a potential role for maternal HLA-G in higher FMc levels in women with SSc.

Human leukocyte antigen-G molecule can be expressed as membrane-bound or soluble form through 7 isoforms generated by alternative splicing: HLA-G1 to G7. HLA-G1 to G4 are membrane bound; only HLA-G1 can be shed from the membrane and also released as sHLA-G1 (29). HLA-G5, G6, and G7 are only soluble forms, HLA-G5 being the most expressed. HLA-G protein expression seems to be modulated by several genetic variations within the coding sequence, the 5′ upstream regulatory region (5′URR) and the 3′ untranslated region (3′UTR) of the *HLA-G* gene (30–37). Conflicting results for sHLA-G production were reported for the most studied 3′UTR polymorphism, the 14-bp insertion/deletion polymorphism in the exon 8 (31, 38–40).

Thus, in the current study we test whether 1/women with SSc display lower sHLA-G levels in their plasma than healthy age-matched women, 2/quantities of persistent fetal Mc in their blood inversely correlate with sHLA-G levels, and 3/sHLA-G levels correlate with *HLA-G* polymorphisms/haplotypes. We recruited 96 healthy women and 106 women with SSc, quantified sHLA-G in plasma samples from respectively 88 and 74 women by enzyme linked immunosorbent assay (ELISA) and analyzed 8 polymorphisms in the 5′URR and 3′UTR of the *HLA-G* gene, including the most described 14-bp insertion/deletion to determine UTR1-8 haplotypes. In parallel, using DYS14 real-time PCR assays, we analyzed male microchimerism of fetal origin in peripheral blood samples from 58 women who had given birth to at least one male child.

## Materials and Methods

### Subjects

A total of 106 women with SSc were recruited in four French hospitals (*St. Louis* hospital in Paris; *Claude Huriez* Hospital in Lille; *La Conception* and *Nord* Hospital in Marseille, France). All patients met the requirements of LeRoy for SSc (41), with 48 having diffuse cutaneous disease (dcSSc) and 55 limited cutaneous disease (lcSSc). Three of them were only defined as having SSc with no indication of clinical subtype. Women were majorly Caucasian (80.2%), then African (14.2%), and Asian (5.6%). Their mean age at blood draw was 54.4 years old [range:16–75].

In parallel, 96 healthy women with no family history of autoimmune disease were recruited in the Centre d’Examen de Santé de l’Assurance Maladie (CESAM), Marseille, France. They were majorly Caucasian (94.8%), then African (3.1%), and Asian (2.1%). Their mean age at blood draw was 50.8 years old [range: 36–69]. Questionnaires with detailed information about history of source of male Mc (transfusion, history of pregnancy, and older brother) were filled in for each participant of the study. For one patient, we could not obtain all information.

### Ethics Statement

This study has received the approval from the French Ethical Committee Marseille 2 and is registered at the INSERM (Biomedical Research Protocol number RBM-04-10). All participants signed written consent forms according to the Declaration of Helsinki (42).

### sHLA-G Measurement

Plasma samples from 88 healthy women and 74 women with SSc were obtained after gradient centrifugation of whole peripheral blood on Ficoll–Hypaque 1077 (Sigma-Aldrich, St. Louis, MO, USA). Plasma were stored at −40°C until tested. Measurement of both shed HLA-G1 and sHLA-G5 isoforms was performed in duplicate on plasma samples from 88 healthy women and 74 women with SSc using the ELISA assay kit (EXBIO/Biovendor, Karásek, Czech Republic; capture antibody: MEM-G/9), defined at the “Wet-Workshop for the Quantification of sHLA-G” in 2004 (43) according to the manufacturer’s instructions. It is to note that the current assay does not allow to distinguish which isoform HLA-G1 or G5 is the most expressed. sHLA-G standard was diluted to obtain a calibrator curve within a range from 3.91 to 125 International units/mL (IU/mL) for sHLA-G ELISA. The total protein concentration levels were expressed in IU/mL of plasma.

### DNA Sample Handling

DNA from 96 healthy women and 106 women with SSc was isolated from 350 µl of blood samples by commercially available method (Qiagen EZ1). DNA concentrations were quantified by Bio-drop instrument according to the manufacturer’s protocol and further by beta-globin-specific Q-PCR as previously described (44). DNA samples from 58 women who had given birth to at least 1 male child were further analyzed for microchimerism studies (38 SSc and 20 healthy women). All DNA samples were sent to the Immunogenetic laboratory at the French Blood Transfusion Department (EFS), Marseille, France, for analyzing the 5′URR and 3′UTR polymorphisms (see below).

### Quantification of Male Mc of Fetal Origin

Male Mc was quantified by a standardized real-time PCR for a Y-chromosome-specific sequence DYS14 on a Light Cycler^®^ with Light Cycler^®^ Fast Start DNA Master PLUS Reaction kits (Roche, Indianapolis, IN, USA) as previously described (12). Sensitivity of the DYS14 assay was of 1 genome equivalent (1 gEq) of male cell in a background of 20,000 gEq female cells. Each DNA sample from whole peripheral blood was then tested for DYS14 amplification in 10 aliquots of DNA equivalent of 20,000 cells (=132 ng with the conversion of 1 cell = 6.6 pg). The amount of male DNA was expressed as the number of gEq of male cells per million (M) of gEq of female cells (gEq/M).

There was no occurrence of a positive amplification in any of the negative controls (no DNA template). A triplicate of male DNA at 10 or 50 gEq, in a background of 20,000 gEq, was systematically run in each plate and served as inter-plate reference standard and positive control.

Presence of male DNA has been tested in peripheral blood from 58 women (38 women with SSc and 20 healthy women) who had previously given birth to at least one son. Obstetrical and clinical characteristics are detailed in Tables 1 and 2, respectively.

**Table 1:** Obstetrical and clinical characteristics of women with SSc quantified for male DNA and soluble HLA-G (sHLA-G) levels.

SSc women	Age at blood draw (years)	Disease duration at blood draw (years)	# of sons	Age of last son (years)	Spontaneous or induced abortions	Blood transfusions	# of older brothers	Male Mc^a^ (gEq/M)	sHLA-G^b^ (IU)
SSc01	63	10	1	25	1	Ukn^c^	**0**	**22.5**^d^	84.0
SSc02	60	5	1	35	0	0	**0**	**0.5**	23.6
SSc03	45	2	1	20	0	0	**0**	**12.7**	17.1
SSc04	58	1	1	30	0	0	**1**	**1.6**	49.2
SSc05	73	6	2	Ukn	0	0	0	0	119.7
SSc06	49	5	1	9	1	0	**0**	**4.5**	83.5
SSc07	78	23	1	56	0	0	**0**	**2.6**	83.0
SSc08	64	1	6	Ukn	0	0	1	0.0	62.2
SSc09	55	6	3	33	0	**1**^e^	**0**	**12.5**	25.2
SSc10	45	5	2	Ukn	1	0	**3**	**10.0**	21.3
SSc11	51	4	2	15	1	0	**1**	**4.3**	19.0
SSc12	60	9	1	36	0	0	**1**	**2.7**	116.9
SSc13	62	6	1	37	0	0	1	0.0	52.8
SSc14	47	18	1	16	0	0	**0**	**14.0**	90.8
SSc15	50	5	2	17	0	0	1	0.0	61.3
SSc16	58	5	2	33	0	**4**	**0**	**4.5**	66.2
SSc17	66	9	1	37	3	0	1	0.0	31.0
SSc18	53	3	1	19	1	0	1	0.0	142.0
SSc19	62	23	1	Ukn	1	0	0	0.0	95.9
SSc20	72	6	1	Ukn	2	Ukn	2	0.0	177.2
SSc21	64	5	2	Ukn	0	**1**	1	0.0	176.7
SSc22	35	5	1	10	0	0	2	0.0	123.9
SSc23	48	0	2	20	0	0	3	0.0	87.9
SSc24	43	2	2	Ukn	1	**1**	0	0.0	98.9
SSc25	57	9	2	Ukn	5	**1**	3	0.0	54.2
SSc26	49	12	1	44	Ukn	0	**Ukn**	**9.7**	25.9
SSc27	40	13	1	15	2	0	0	0.0	47.1
SSc28	66	16	2	37	0	0	2	0.0	34.5
SSc29	62	13	1	40	2	Ukn	**0**	**2.3**	67.5
SSc30	61	1	1	32	1	0	**1**	**7.1**	16.3
SSc31	52	2	1	32	0	0	1	0.0	64.2
SSc32	45	22	1	24	0	0	1	0.0	89.2
SSc33	37	0	1	7	0	0	1	0.0	164.0
SSc34	54	17	1	26	0	0	0	0.0	76.3
SSc35	56	12	1	17	1	0	**0**	**53.2**	49.8
SSc36	40	3	1	15	1	0	2	0.0	92.8
SSc37	53	19	2	25	2	0	**0**	**0.5**	37.5
SSc38	75	25	4	33	1	**2**	0	0.0	106.3

*^a^Male microchimerism is expressed in genome equivalent of male cells per million of maternal cells (gEq/M)*.

*^b^Quantities of sHLA-G are expressed in International Units per mL of plasma (IU/mL)*.

*^c^Unknown data are abbreviated with ukn*.

*^d^Positive values for male Mc are in bold*.

*^e^Women who had an history of transfusion are in bold and were excluded from statistical analysis, as well as women with unknown data for blood transfusion*.

**Table 2:** Obstetrical and clinical characteristics of healthy women quantified for male DNA and soluble HLA-G (sHLA-G) levels.

Healthy women (HW)	Age at blood draw (years)	# of sons	Age of last son (years)	Spontaneous or induced abortions	Blood transfusions	# of older brothers	Male Mc^a^ (gEq/M)	sHLA-G^b^ (IU)
HW01	37	1	4	0	0	**0**	**0.61**^c^	119.7
HW02	57	2	23	1	0	0	0	26.2
HW03	58	1	22	0	0	2	0	81.2
HW04	58	1	40	3	**1**^d^	**2**	**2.3**	140.7
HW05	42	1	15	2	0	0	0	112.1
HW06	38	1	9	0	0	0	0	13.5
HW07	60	1	32	1	0	0	0	117.8
HW08	54	1	26	0	0	0	0	150.4
HW09	53	1	38	0	0	3	0	37.3
HW10	66	1	35	0	0	0	0	65.0
HW11	47	1	23	0	0	3	0	212.9
HW12	54	2	21	1	0	0	0	157.4
HW13	40	2	9	0	0	0	0	49.3
HW14	54	1	25	0	**2**	1	0	170.2
HW15	46	1	17	1	0	1	0	35.6
HW16	49	1	24	0	0	0	0	94.6
HW17	41	2	13	1	0	**1**	**1.07**	60.0
HW18	50	1	19	1	0	0	0	76.5
HW19	59	3	25	2	0	0	0	62.2
HW20	49	1	22	1	0	1	0	228.0

*^a^Male microchimerism is expressed in genome equivalent of male cells per million of maternal cells (gEq/M)*.

*^b^Quantities of sHLA-G are expressed in International Units per mL of plasma (IU/mL)*.

*^c^Positive values for male Mc are in bold*.

*^d^Women who had an history of transfusion are in bold and were excluded from statistical analysis*.

### *HLA-G* 5′URR and 3′UTR Genotyping and UTR Haplotype Estimation

A home-made primer extension method, as previously described, was used to simultaneously analyze four SNPs in the 5′URR region (−725C/G/T rs1233334, −716 G/T rs2249863, −201 G/A rs1233333, and −56C/T rs17875397) and four polymorphisms in the 3′UTR region (ins/del exon 8 rs66554220; 3142C/G rs1063320; 3187G/A rs9380142; and 3196C/G rs1610696) (45). *HLA-G* genotypes were analyzed using GeneMapper 4.0 with specific detection parameters. UTR~*HLA-G* haplotypes were estimated using an EM algorithm from the Gene (46) program and confirmed using the EM and ELB algorithms from the Arlequin v3.5.1.2 package. Data were analyzed and interpreted as previously described (45, 47).

### Statistical Analyses

Mann–Whitney test was applied to compare sHLA-G dosages (expressed in IU/mL) between patients and controls, independently or according to *HLA-G* genetic status, SSc-associated treatments, age of probands or disease duration.

To quantify the degree to which sHLA-G quantities is related to fetal male DNA quantities, we excluded women with history of blood transfusion, or for whom we did not have transfusion information to not confound natural (arising from pregnancy) and iatrogenic (from transplantation) sources of male Mc. Nevertheless, to test whether male DNA levels arising from pregnancy correlated with sHLA-G levels in plasma, we did not eliminate women who had spontaneous or induced abortions additionally to their son, although we did not know the sex of the fetus. Correlation was assessed with Spearman rank correlation test. *P* values less than 0.05 were considered significant. Statistical analyses were conducted using GraphPad Prism 6 (La Jolla, CA, USA).

Significant deviations from expected values at Hardy–Weinberg equilibrium (HWE) were tested for each polymorphisms using Chi square “goodness of fit” test (http://vassarstats.net). As for two SNPs HWE was not respected (−201 (A/G), −56 (C/T)) in the healthy population, and numbers were small, we did not use the classical χ^2^ for evaluating whether women with SSc were less often heterozygous for *HLA-G* polymorphisms than healthy women, but rather the Bayesian statistical method (R software version 3.0.2.10), as recommended in such cases (48). *P* values were further corrected for multiple comparison tests by Benjamini–Hochberg correction (49).

Chi-square “χ^2^” tests with Benjamini–Hochberg corrections were used to compare UTR haplotype frequencies between healthy women and women with SSc.

## Results

### Women With SSc Displayed Lower sHLA-G in Their Plasma Compared to Healthy Women

**Figure 1:**
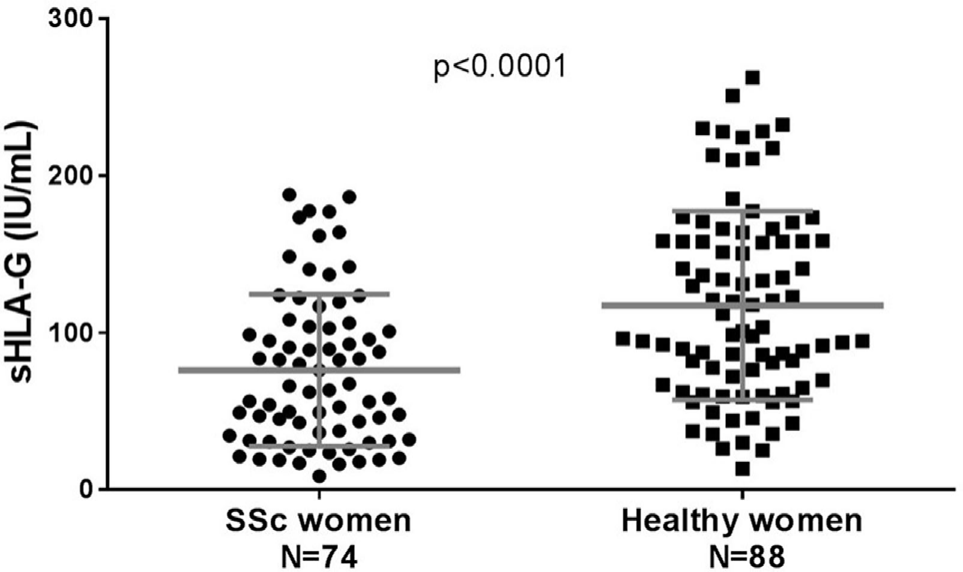
Quantification of soluble HLA-G (sHLA-G) in plasma from healthy and SSc women. Quantification of sHLA-G is expressed in International Units per mL of plasma (IU/mL). Black circles represent values for women with SSc and black squares represent values for healthy women. Means and SDs are given for each scatter plot by gray bars. *P* value is calculated by Mann–Whitney test.

Soluble HLA-G (shed HLA-G1 and sHLA-G5) could be detected by the ELISA technique defined at the “Wet-Workshop for the Quantification of sHLA-G” in all plasma samples (43), with quantities ranging from 8.8 International Units (IU) per mL of plasma to 187.9 IU/mL in samples from women with SSc and from 13.5 to 262.5 IU/mL in samples from healthy women (Figure 1).

Women with SSc had significantly lower quantities of sHLA-G than healthy women in their plasma with respectively a mean of 76.2 IU/mL compared to 117.5 IU/mL (*p* < 0.0001).

### Lower sHLA-G Was Independent of Clinical Subtypes, Autoantibody Profiles, Disease Duration, Treatments, or *HLA-G* 5′URR/3′UTR Polymorphisms

**Figure 2:**
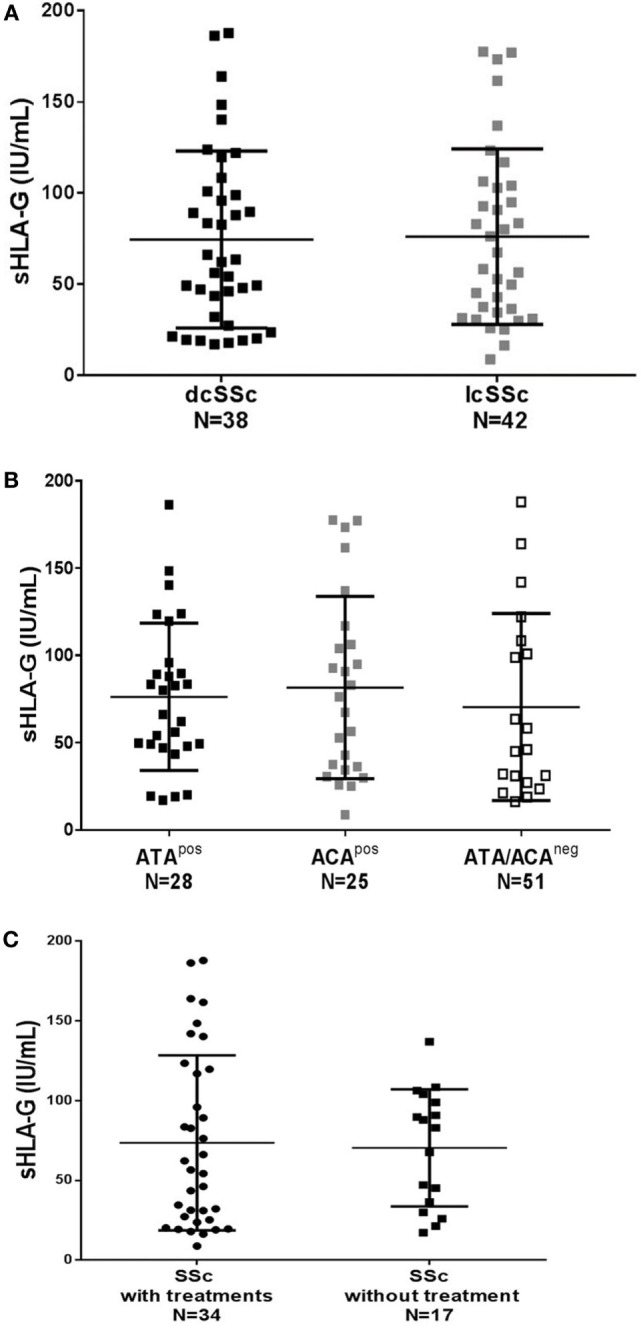
Quantification of soluble HLA-G (sHLA-G) in plasma from women with SSc divided according to **(A)** their clinical status: black squares represent values for women with diffuse cutaneous SSc (dcSSc), gray squares represent values for women with limited cutaneous SSc (lcSSc); **(B)** their autoantibody status: black squares represent values for women with SSc positive for anti-topoisomerase antibodies (ATA^pos^), gray squares represent values for women with anti-centromere antibodies (ACA^pos^), and white squares represent values for women negative for both autoantibodies (ATA/ACA^neg^); and **(C)** their treatments: black circles represent values for women who are under immunosuppressive therapies and/or anti-inflammatory medications, labeled “with treatments”; black squares represent values for women who have only drugs to treat consequences of the disease and labeled “without treatments.” Means and SDs are given for each scatter plot by gray bars *P* values are calculated by Mann–Whitney test in **(A,C)** and one way-ANOVA test in **(B)** and are not significant for any of the graphs (*p* = 0.85, *p* = 0.49, and *p* = 0.75, respectively).

Quantities of sHLA-G were identical whether women with SSc had diffuse or limited cutaneous SSc (Figure 2A), were positive for ATA or anti-centromere antibodies (ACA) or were negative for both autoantibodies (Figure 2B). No correlation was observed between sHLA-G levels and the age of probands or disease duration (data not shown).

Soluble HLA-G dosages were not different whether women were under immunosuppressive therapies (cyclophosphamide, methotrexate, etc.) with or without anti-inflammatory medications (non-steroidal or corticosteroids, etc.) or had only drugs to treat consequences of the disease, such as vasodilatator therapies (calcium channel blockers, angiotensin converting enzyme inhibitors, etc.), antifibrotic agents (colchicine, etc.), or others (i.e., proton pump inhibitors for gastroesophageal reflux) (Figure 2C).

**Figure 3:**
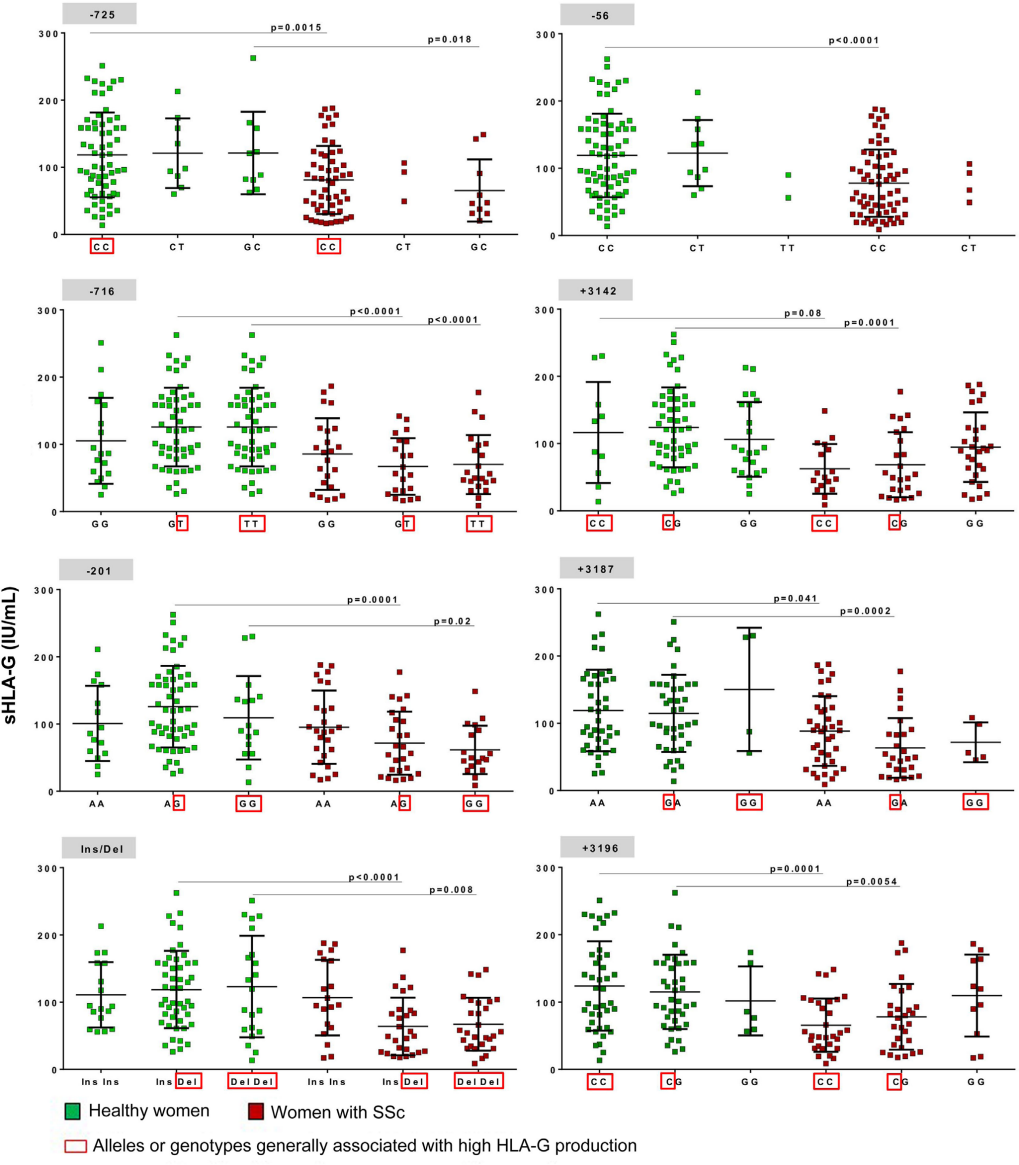
Concentration of soluble HLA-G (sHLA-G) per mL of plasma according to different human leukocyte antigen (HLA)-G genotypes. The eight polymorphisms tested (−725, −716, −201, −56, Ins/Del, +3,142, +3,187, and +3,196) are represented with their three possible genotypes (i.e., GG, GT, and TT for −716 SNP) except for −725 SNP for which six genotypes exist: CC, CT, GC, TT, GG, and GT but the last three were not represented on the graph as only few women had these genotypes; sHLA-G concentration values are represented for each genotypes in green (on the left side) for healthy women and in red (on the right side) for women with SSc. A red square is surrounding alleles or genotypes having been described in most studies with high HLA-G production. Means and SDs are given for each scatter plot by black bars. *P* values are calculated by Mann–Whitney tests.

Finally, the significant decrease of sHLA-G expression observed in SSc women compared to healthy women seemed independent from the eight *HLA-G* 5′URR/3′UTR tested polymorphisms as the difference remained significant when patients and controls were separated according to their *HLA-G* genotypes for each polymorphic site (Figure 3).

### Lower sHLA-G Expression Correlated With Higher Levels of Male Microchimerism

**Figure 4:**
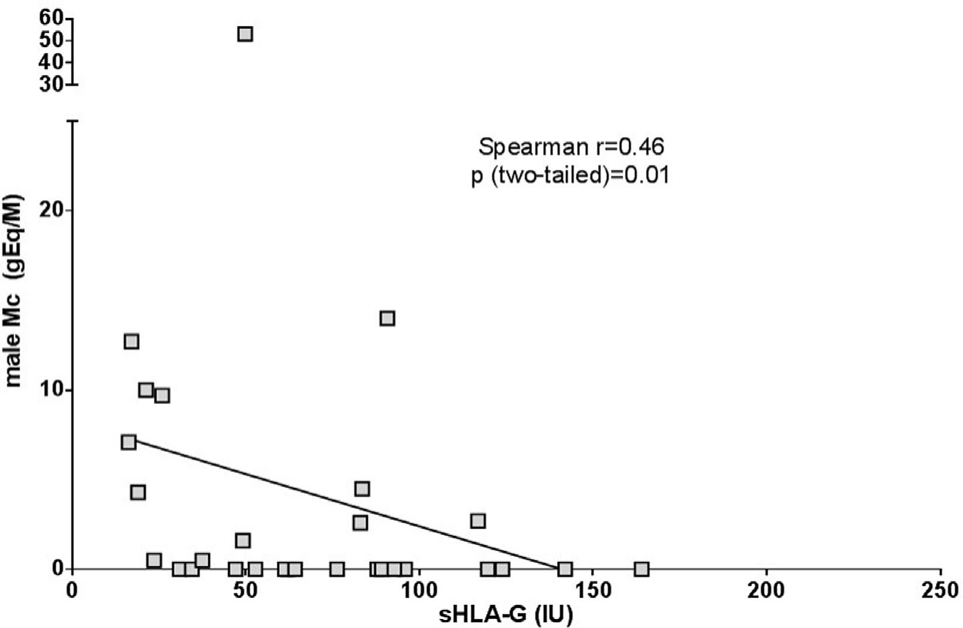
Correlation between male Mc quantities and soluble HLA-G (sHLA-G) levels in women with SSc. On the *x*-axis are represented values for sHLA-G expressed in International Units per mL of plasma (IU/mL). On the *Y*-axis are represented the genome equivalent of male microchimeric cells per million of mother’s cells (gEq/M). Correlation is assessed with Spearman rank correlation test.

In women with SSc, sHLA-G expression was inversely correlated with levels of fetal Mc in their peripheral blood as illustrated in Figure 4 [Spearman correlation coefficient *r* = −0.46, 95% confidence interval (−0.71 to −0.10), *p* = *0.01*]. Quantities of male DNA in maternal blood seemed to be independent from the number of sons, the number of years since the birth of last son or the disease duration (Table 1). Male Mc observed here is more probably from pregnancy than from an older brother, since 69% of women with SSc who had male Mc did not have an older brother (Table 1).

### Women With SSc Displayed a Lack of Heterozygosity for *HLA-G* Polymorphisms Compared to Healthy Women

A lack of heterozygosity among genotypes was observed in the SSc population with statistical significance for four polymorphisms: −716 (G/T), −716 (G/T), −201 (G/A), exon 8 (Ins/Del), and +3,142 (G/C), even after correction for multiple comparisons (Table 3). However, none of the polymorphisms described here displayed allelic frequency differences between women with SSc and healthy women.

**Table 3:** Genotype and allele frequencies of eight human leukocyte antigen-G (HLA-G) polymorphisms in healthy and SSc women.

HLA-G polymorphisms	Genotypes or alleles	Healthy women *N* = 96		SSc women *N* = 106		*P* values^a^ cases vs controls
		*N*	%	*N*	%	
	CC	68	70.8	76	71.7	
	**CT**^b^	10	10.4	8	7.5	
	**GC**	13	13.5	20	18.9	
	TT	3	3.1	0	0.0	***ns***^c^
**−725**	GG	1	1.0	2	1.9	
	**GT**	1	1.0	0	0.0	
	
	C	159	82.8	180	84.9	
	G	16	8.3	24	11.3	***ns***
	T	17	8.9	8	3.8	

	GG	19	19.8	37	34.9	
	**GT**	**57**	**59.4**	**37**	**34.9**	***P*** = ***0.0003***
**−716**	TT	20	20.8	32	30.2	***Pc*** = ***0.002***
	
	G	95	49.5	116	54.7	***ns***
	T	97	50.5	96	45.3	

	AA	16	16.7	35	33.0	
	**GA**	**60**	**62.5**	**45**	**42.5**	***P*** = ***0.002***
**−201**	GG	20	20.8	26	24.5	***Pc*** = ***0.006***
	
	A	92	47.9	115	54.2	***ns***
	G	100	52.1	97	45.8	

	CC	82	85.4	98	92.5	
	CT	11	11.5	8	7.5	***ns***
**−56**	TT	3	3.1	0	0.0	
	
	C	175	91.1	204	96.2	***ns***
	T	17	8.9	8	3.8	

	Ins Ins	18	18.8	25	23.6	
	**Ins Del**	**54**	**56.3**	**42**	**39.6**	***P*** = ***0.01***
**Ins/Del**	Del Del	24	25.0	39	36.8	***Pc*** = ***0.02***
	
	Ins	90	46.9	92	43.4	***ns***
	Del	102	53.1	120	56.6	

	CC	13	13.5	24	22.6	
	**GC**	**57**	**59.4**	**41**	**38.7**	***P*** = ***0.002***
**+3,142**	GG	26	27.1	41	38.7	***Pc*** = ***0.008***
	
	C	83	43.2	89	42.0	***ns***
	G	109	56.8	123	58.0	

	AA	46	47.9	60	56.6	
	GA	46	47.9	41	38.7	***ns***
**+3,187**	GG	4	4.2	5	4.7	
	
	A	138	71.9	161	75.9	***ns***
	G	54	28.1	51	24.1	

	CC	47	49.0	44	41.5	
	CG	43	44.8	48	45.3	***ns***
**+3,196**	GG	6	6.3	14	13.2	
	
	C	137	71.4	136	64.2	***ns***
	G	55	28.6	76	35.8	

*^a^*P* values (*P*) are calculated by Bayesian tests and *Pc* (*P* corrected) values correspond to multiple comparison correction by Benjamini–Hochberg test (see Materials and Methods)*.

*^b^Genotypes in bold are heterozygous, comparisons are made between heterozygous polymorphisms and homozygous polymorphisms (i.e., for −716 (G/T): GT vs GG + TT)*.

*^c^Non significant p values are noted ns*.

As there was significantly more individuals of African and Asian origin in cases than in controls (*P* < 0.01), we performed the same analysis by considering only Caucasian individuals. Results were similar than the ones with all cases and controls (Table S1 in Supplementary Material).

### Frequencies of UTR Haplotypes in Healthy Women and Women With SSc

*Human leukocyte antigen-G* haplotypes were defined by four SNPs in the 5′URR and four polymorphisms in the 3′UTR are named UTR. UTR frequencies between women with SSc and healthy women (Table 4), did not display any significant difference, although a tendency for an increase of the UTR2 haplotype in SSc, particularly in dcSSc was observed, none of the *p* values remained significant after correction for multiple tests. Similarly, the small decrease for the UTR5 and UTR7 haplotypes in SSc, particularly in dcSSc, did not remain significant after correction.

**Table 4:** Frequencies of UTR haplotypes in healthy women and women with SSc.

UTR	Coding allele	5′URR					3′UTR			Haplotype number, frequency (%)			
		
	Human leukocyte antigen-G	−725	−716	−201	−56	*14 bp*	3,142	3,187	3,196	**Healthy women, *N* = 96** ***192 hapl***.	**SSc women, *N* = 106** ***212 hapl***.	**dcSSc women, *N* = 48** ***96 hapl***.	**lcSSc women, *N* = 55** ***110 hapl***.
**UTR-1**	**01:01**	**C**	**T**	**G**	**C**	***Del***	**C**	**G**	**C**	**51 (26.6)**^a^	**50 (23.6)**	**24 (25.0)**	**25 (22.7)**
**UTR-2**	**All**	**C**	**G**	**A**	**C**	***Ins***	**G**	**A**	**G**	**55 (28.6)**	**76 (35.8)**	**40 (41.7)^a^**	**34 (30.9)**
	**01:01**									38 (19.8)	57 (26.9)	31 (32.3)	25 (22.7)
	**01:05N**									2 (1.0)	4 (1.9)	1 (1.0)	2 (1.8)
	**01:06**									15 (7.8)	15 (7.1)	8 (8.3)	7 (6.4)
**UTR-3**	**01:04**	**C**	**G**	**A**	**C**	***Del***	**G**	**A**	**C**	**19 (9.9)**	**31 (14.6)**	**11 (11.5)**	**19 (17.3)**
**UTR-4**	**01:01**	**G**	**T**	**G**	**C**	***Del***	**C**	**A**	**C**	**16 (8.3)**	**24 (11.3)**	**11 (11.5)**	**12 (10.9)**
**UTR-5**	**01:03**	**T**	**T**	**G**	**T**	***Ins***	**G**	**A**	**C**	**17 (8.9)**	**8 (3.8)**^b^	**2 (2.1)**^c^	**6 (5.5)**
**UTR-6**	**01:01**	**C**	**T**	**G**	**C**	***Del***	**C**	**A**	**C**	**13 (6.8)**	**14 (6.6)**	**4 (4.2)**	**9 (8.2)**
**UTR-7**	**01:01**	**C**	**G**	**A**	**C**	***Ins***	**G**	**A**	**C**	**18 (9.4)**	**8 (3.8)**	**3 (3.1)**^d^	**5 (4.5)**
**UTR-8**	**01:01**	**C**	**G**	**G**	**C**	***Del***	**C**	**G**	**C**	**3 (1.6)**	**1 (0.5)**	**1 (1.0)**	**0 (0)**

*The polymorphic sites presented are in 5′URR at position −725, −716, −201, −56, and in 3′UTR, the 14 bp insertion/deletion, +3,142, +3,187, and +3,196. The most frequent *human leukocyte antigen-G* haplotypes considering the mRNA sequences, named according to Castelli et al. (33), are represented (UTR1–UTR8)*.

*^a^For each subgroup of women tested, the first value represents the number of a given haplotype and the value between brackets indicates the frequency of this haplotype. All *P* values to compare UTR frequencies from healthy women to UTR frequencies from SSc women are calculated by χ^2^ test. Corrections for multiple comparisons are done by Benjamini–Hochbergtest (Pc)*.

*^a^P = 0.02, Pc = not significant*.

*^b^P = 0.034, Pc = not significant*.

*^c^P = 0.017, Pc = not significant*.

*^d^P = 0.040, Pc = not significant*.

## Discussion

The HLA-G molecule has tolerogenic functions and its role has been described in transplantation, human reproduction, and more recently in some rheumatologic diseases, including SSc [for review see Ref. (50)]. In the present study, we focus on the initially described role of HLA-G expression on immune tolerance during pregnancy. We hypothesized that the observation described by our group and others, that parous women with SSc maintain higher levels of fetal Mc compared to matched healthy controls, is due to an impaired expression of maternal sHLA-G. Studies on preeclampsia (PE) support our hypothesis as women with pregnancies complicated with PE have lower levels of sHLA-G in their blood in the second and third trimester compared to controls [for review see Ref. (51)]. In parallel to low sHLA-G production, pregnancies with PE are associated with higher passage of circulating fetal DNA and fetal cells in maternal peripheral blood (24, 25).

In the current study, we validate the hypothesis that women with SSc produce less sHLA-G in their plasma than healthy women. Quantities are low and at comparable levels whether women with SSc have a dcSSc or lcSSc and whether they produce ATA, ACA, or none of those antibodies. Notably, treatments associated with SSc do not seem to have an influence on sHLA-G levels, as women who had only drugs treating consequences of the disease (i.e., gastroesophageal reflux, etc.) have levels as low as women under immunosuppressive therapies and/or anti-inflammatory medications. The ELISA used is a sandwich enzyme immunoassay for the quantitative measurement of any soluble forms of HLA-G. Therefore, it measures the shed sHLA-G1 and the secreted HLA-G5 isoform without distinction and we cannot tell at this point which one of the isoform is less present in our measurements.

To our knowledge, only two studies have recently evaluated sHLA-G in sera/plasma samples from patients with SSc (52, 53). The first one showed a tendency for lower levels in patients but failed to find statistical difference. The second one showed a significant increased expression in patients. Contradictory results could come from the relatively small number of patients [*N* = 35 in Ref. (53)] and/or of healthy controls tested in both studies [*N* = 32 and 40, respectively (52, 53)].

Although several *HLA-G* polymorphisms have been described as being associated with reduced production of sHLA-G, we could not find any association with *HLA-G* genetic polymorphism and SSc or sHLA-G expression in women with SSc. Our results are concordant with those of Nilsson et al. describing no association with *HLA-G* haplotype or with ins/del polymorphism with increased risk of developing severe preeclampsia/eclampsia in a cohort of more than 900 women (54). Nevertheless, women with PE have low levels of sHLA-G as reported in several studies (20, 55).

As our study is the first to test these polymorphisms in SSc (except Ins/Del), it would be of great value to confirm our data on large cohorts of patients with SSc and on other ethnic groups. Nevertheless, our data suggest that the eight polymorphisms tested in the 5′URR and in the 3′UTR are not sufficient to predict any decreased production of sHLA-G observed in SSc; other SNPs in regulatory regions might contribute to sHLA-G expression modulation. DNA methylation is an important mechanism for regulating gene expression and a few reports suggest that methylation of the *HLA-G* promoter is associated with lower secretion of HLA-G in women with preeclampsia (56, 57). Moreover, our results showing that women with SSc who carry *HLA-G* alleles commonly associated with high sHLA-G levels have low sHLA-G levels, comfort a possible epigenetic regulation of sHLA-G production on 5′ URR or 3′UTR.

Although patients did not have a particular *HLA-G* allele over-represented, they were statistically more often homozygous than heterozygous for *HLA-G* polymorphism genotypes (i.e., Ins/Ins or Del/Del > Ins/Del). High heterozygosity for MHC genes is classically observed in the general healthy population and may be enforced by natural selection for a higher genetic diversity (58). On the opposite homozygotic carriers could be less resistant to broader spectrum of pathogens as they have less antigen presentation possibilities than heterozygotic carriers. A recent study showed that women homozygous for −716 *HLA-G* SNP are at higher risk for spontaneous abortion (59). This confirms the disadvantage of carrying homozygous *HLA-G* polymorphisms for success of reproduction and may be related to the consequence that women with pregnancy complications are at higher risk to develop later SSc.

Besides the role of HLA-G in pregnancy, several studies have indicated a wider immunoregulatory role of this molecule (60). In this context, the expression of HLA-G in inflammatory and rheumatologic diseases is a relatively recent research area. Our results open a new field of investigation for sHLA-G regulation in scleroderma. Finding the pathways explaining low production in women with SSc may also conduct to a better understanding for higher persistence of fetal Mc. HLA-G regulation is likely a key factor for FMc passage and maintenance. Animal models are now required as a proof of concept that maternal sHLA-G molecules play a role on higher traffic of fetal cells during pregnancy.

## Ethics Statement

This study has received the approval from the French Ethical Committee Marseilles 2 and is registered at the INSERM (Biomedical Research Protocol number RBM-04-10). All participants signed written consent forms according to the Declaration of Helsinki (42).

## Author Contributions

JC, KK, CP, and NL conceived and designed the experiments. JC, KK, SK, DA, MH, and LH performed the experiments. JC, KK, EP, CP, and NL analyzed the data. DF-B, BG, JH, EH, and JR contributed to patient and control recruitments. JC, KK, CP, and NL wrote the paper.

## Conflict of Interest Statement

The authors declare that the research was conducted in the absence of any commercial or financial relationships that could be construed as a potential conflict of interest.
