# Supplementary material for: Soluble HLA-G Expression Inversely Correlates With Fetal Microchimerism Levels in Peripheral Blood From Women With Scleroderma
Source: Front Immunol. 2018 Aug 14;9:1685. doi: 10.3389/fimmu.2018.01685 (PMC6104483; doi:10.3389/fimmu.2018.01685)
Supplement: Supplementary file 1 [file Table_1.docx]

Supplementary Table S1 : Genotype and allele frequencies of eight HLA-G polymorphisms in healthy and SSc Caucasian women.

| **HLA-G** | **Genotypes** | **Caucasian Healthy women** | | **Caucasian SSc women** | | **P values^a^** | |
| --- | --- | --- | --- | --- | --- | --- | --- |
| **polymorphisms** | **or alleles** | **N=91** | | **N=85** | | **Cases vs Controls** | |
|  |  | **N** | **%** | **N** | **%** |  |  |
|  | CC | 64 | 70,3 | 60 | 70,6 |  |  |
|  | **CT**^b^ | 9 | 9,9 | 8 | 9,4 |  |  |
|  | **GC** | 13 | 14,3 | 15 | 17,6 |  |  |
|  | TT | 3 | 3,3 | 0 | 0,0 | ***ns*^c^** |  |
|  | GG | 1 | 1,1 | 2 | 2,4 |  |  |
| **-725** | **GT** | 1 | 1,1 | 0 | 0,0 |  |  |
|  | C | 150 | 82,4 | 143 | 84,1 |  |  |
|  | G | 16 | 8,8 | 19 | 11,2 | ***ns*** |  |
|  | T | 16 | 8,8 | 8 | 4,7 |  |  |
|  | GG | 16 | 17,6 | 30 | 35,3 |  |  |
|  | **GT** | **55** | **60,4** | 30 | **35,3** | ***p= 0.004*** |  |
| **-716** | TT | 20 | 22,0 | 25 | 29,4 | ***pc=0.003*** |  |
|  | G | 87 | 47,8 | 90 | 52,9 | ***ns*** |  |
|  | T | 95 | 52,2 | 80 | 47,1 |  |  |
|  | AA | 14 | 15,4 | 29 | 34,1 |  |  |
|  | **GA** | **57** | **62,6** | 35 | **41,2** | ***p=0.002*** |  |
| **-201** | GG | 20 | 22,0 | 21 | 24,7 | ***pc=0.006*** |  |
|  | A | 85 | 46,7 | 93 | 54,7 | ***ns*** |  |
|  | G | 97 | 53,3 | 77 | 45,3 |  |  |
|  | CC | 78 | 85,7 | 77 | 90,6 |  |  |
|  | CT | 10 | **11,0** | 8 | 9,4 | ***ns*** |  |
| **-56** | TT | 3 | 3,3 | 0 | 0,0 |  |  |
|  | C | 166 | 91,2 | 162 | 95,3 | ***ns*** |  |
|  | T | 16 | 8,8 | 8 | 4,7 |  |  |
|  | Ins Ins | 17 | 18,7 | 23 | 27,1 |  |  |
|  | **Ins Del** | **51** | **56,0** | 32 | **37,6** | ***p=0.007*** |  |
| **Ins/Del** | Del Del | 23 | 25,3 | 30 | 35,3 | ***pc=0.015*** |  |
|  | Ins | 85 | 46,7 | 78 | 45,9 | ***ns*** |  |
|  | Del | 97 | 53,3 | 92 | 54,1 |  |  |
|  | CC | 13 | 14,3 | 19 | 22,4 |  |  |
|  | **GC** | **55** | **60,4** | 31 | **36,5** | ***p=0.0008*** |  |
| **+3142** | GG | 23 | 25,3 | 35 | 41,2 | ***pc=0.003*** |  |
|  | C | 81 | 44,5 | 69 | 40,6 | ***ns*** |  |
|  | G | 101 | 55,5 | 101 | 59,4 |  |  |
|  | AA | 43 | 47,3 | 48 | 56,5 |  |  |
|  | GA | 44 | **48,4** | 33 | 38,8 | ***ns*** |  |
| **+3187** | GG | 4 | 4,4 | 4 | 4,7 |  |  |
|  | A | 130 | 71,4 | 129 | 75,9 | ***ns*** |  |
|  | G | 52 | 28,6 | 41 | 24,1 |  |  |
|  | CC | 45 | 49,5 | 34 | 40,0 |  |  |
|  | CG | 40 | **44,0** | 38 | 44,7 | ***ns*** |  |
| **+3196** | GG | 6 | 6,6 | 13 | 15,3 |  |  |
|  | C | 130 | 71,4 | 106 | 62,4 | ***ns*** |  |
|  | G | 52 | 28,6 | 64 | 37,6 |  |  |

^a^P values (P) are calculated by Bayesian tests and Pc (P corrected) values correspond to multiple comparison correction by Benjamini-Hochberg test (see methods). ^b^Genotypes in bold are heterozygous, comparisons are made between heterozygous polymorphisms and homozygous polymorphisms (i.e. for -716 (G/T): GT vs GG +TT). ^c^Non significant p values are noted *ns*.
